# Writing as cognitive rehabilitation in MCI and dementia: a systematic review of therapeutic benefits and applications

**DOI:** 10.3389/fneur.2025.1568336

**Published:** 2025-09-02

**Authors:** Arman Hajikarim-Hamedani, Setareh Rassa, Maryam Noroozian, Delaram Jafari

**Affiliations:** ^1^Cognitive Neurology, Dementia and Neuropsychiatry Research Center, Tehran University of Medical Sciences, Tehran, Iran; ^2^Faculty of Medicine, Tehran Medical Sciences, Islamic Azad University, Tehran, Iran; ^3^Cell and Molecular Biology Department, Tehran Medical Sciences, Azad University, Tehran, Iran

**Keywords:** writing-based therapies, cognitive rehabilitation, mild cognitive impairment (MCI), Handwriting, dementia

## Abstract

**Background:**

Dementia, a worldwide health issue characterized by cognitive and functional deterioration, requires effective non-pharmacological interventions. Writing-based therapies, including Handwriting and typing, enhance memory, attention, and executive functions, providing cognitive, emotional, and social advantages. This systematic review examines the changing role of writing in dementia rehabilitation.

**Methods:**

In accordance with PRISMA guidelines, studies from PubMed, Scopus, and Web of Science (1991–2024) were examined. Data were extracted utilizing EndNote 21, concentrating on demographics, and effects of writing. Methodological quality was evaluated employing modified Cochrane and Effective Public Health Practice Project instruments.

**Results:**

The systematic review highlights writing-based interventions, such as journaling, poetry, Chinese calligraphy, and memory notebooks, as effective instruments for cognitive, emotional, and social rehabilitation in mild cognitive impairment (MCI) and dementia. These techniques improve memory, identity, emotional control, and cognitive resilience, while cultural and multimodal approaches provide supplementary advantages.

**Conclusion:**

This review emphasizes writing as an accessible and culturally appropriate therapeutic approach for cognitive rehabilitation in dementia. Calligraphy activities combine memory, motor skills, and concentration, providing cost-effective tools to improve mental and emotional health while fostering cultural connection and engagement.

## Graphical Abstract

**Figure:**
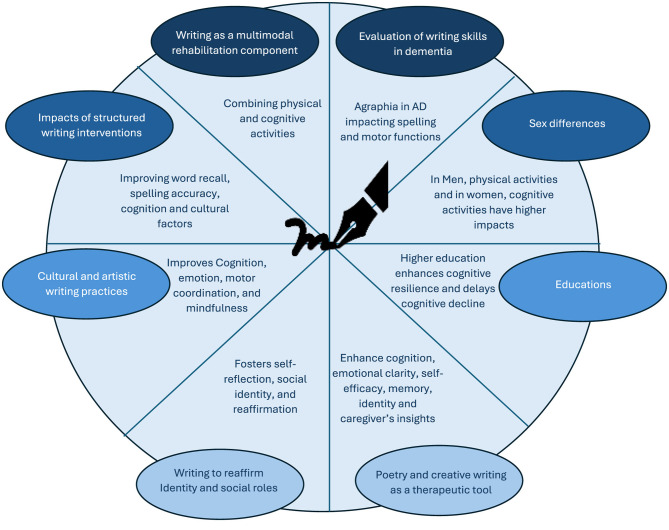

## 1 Introduction

Dementia is a progressive neurological disease that affects millions of people worldwide, resulting in considerable cognitive, emotional, and functional loss (1). It is a significant global health challenge, with its prevalence anticipated to rise substantially in the following decades due to the aging population. It is marked by cognitive deterioration, encompassing memory deficits, communication challenges, and diminished quality of life, which profoundly impacts patients and their families. Due to the constraints and side effects of pharmacological therapies, patients become non-compliant with taking their medications, which worsens their clinical symptoms and the response to the medications (2, 3). Therefore non-pharmacological interventions have risen to enhance quality of life, wellbeing and reduce behavioral and psychological symptoms of dementia (4–6).

Artistic interventions have been proven to have great potential for improving cognitive functions and mental health in patients with mild cognitive impairment (MCI) or Dementia (6). Art therapies can stimulate cognition through various pathways, including stimulation of the temporal lobe, affecting recognition and expression using language, and the parietal lobe, improving the spatial position and fine motor functions (7), training the hand-brain interactions and maintaining motor skills and coordination (8), and also providing patients with a non-verbal communication which enables them to overcome disparities of self-expression due to impaired language ability (9). For instance, art appreciation, Sculpture making, painting, and drawing have beneficial effects on overall cognition, including attention, concentration, and memory (6). Moreover, among individuals with dementia, singing is associated with improved verbal fluency, executive function, and episodic memory (10), fostering mood enhancement and social connection.

Artistic interventions also improve behavioral and psychological symptoms of dementia (BPSD), such as motivation, mood, apathy, aggressive behavior, agitation, sadness, self-esteem, communication, and overall self-reported wellbeing in patients with dementia (11–15).

Writing-based therapies are types of non-pharmacological interventions that have garnered attention for their ability to increase cognitive function, enhance memory, recall and emotional expression, and facilitate social engagement (16, 17). Further, Van der Weel and Van der Meer (18) have discovered that writing by hand increases the brain connectivity, which has been shown to facilitate learning and memory (19) on the other hand research indicates that early verbal competence correlates with cognitive resilience in later life (20, 21). Thus, sustaining language involvement through activities like journal writing has been linked to a diminished risk of dementia and enhanced cognitive performance (5, 21). In its diverse manifestations, writing serves as a conduit for self-expression while also stimulating memory, attention, and executive functioning, which are frequently impaired in dementia (22). Writing has emerged as a potential rehabilitation approach for dementia patients, providing a unique combination of cognitive, emotional, and social advantages.

In this systematic review, we aimed to gather and highlight the advantages and effects of Handwriting and its therapeutic uses in mild cognitive impairment and dementia rehabilitation. By synthesizing existing evidence, we intend to illustrate how structured writing practices—ranging from reminiscence therapy to creative storytelling—can enhance cognitive and emotional wellbeing in individuals with dementia. This article examines how writing promotes neurocognitive engagement, evaluates the efficacy of writing therapies, and discusses their practical applications in clinical and community contexts.

## 2 Materials and methods

### 2.1 Instrument

**Figure 1:**
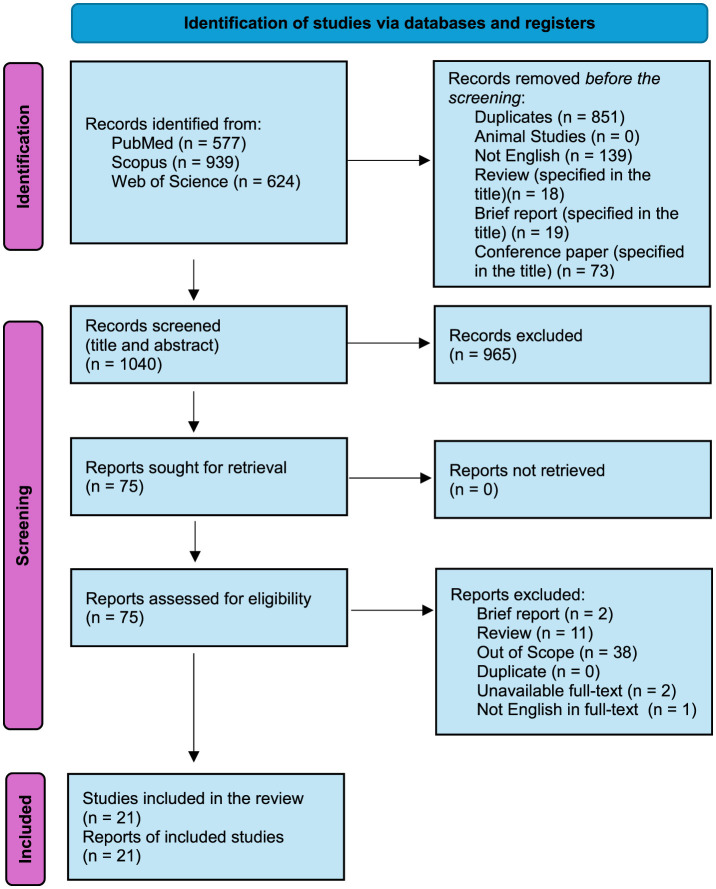
Selection of studies. Preferred Reporting Items for Systematic Reviews and Meta-Analyses (PRISMA) flow diagram. Of the 2,140 identified studies, 21 were included in the synthesis after applying the inclusion and exclusion criteria (24).

We adhered to the recommendations of the Preferred Reporting Items for Systematic Reviews and Meta-Analyses (PRISMA) (23, 24) checklist to carry out a systematic review. The databases selected for this research (Figure 1) were PubMed, Scopus, and Web of Science.

### 2.2 Inclusion and exclusion criteria

The subsequent inclusion and exclusion criteria were defined before the screening. The inclusion criteria were human research only and publications in English. Exclusion criteria included research letters, editorials, reviews, and comments.

### 2.3 Search strategy

Eligible studies were identified using PubMed, Scopus, and Web of Science searches. They were published between January 1, 1991, and April 1, 2024.

Unique search methodologies were formulated for each database. The search terms employed to retrieve data from each thesaurus commenced with amalgamating general terms from cognitive domains and those based on prior knowledge. A sequence of preliminary scoping searches was performed, leading to the identification of several essential search keywords. An illustration of a conclusive search protocol for the PubMed database is presented below.

1. (dementia^*^[Title/Abstract]) OR (dementia^*^[MeSH Terms]) OR (Childhood dementia[Title/Abstract]) OR (Chronic Traumatic Encephalopathy dementia[Title/Abstract]) OR (HIV associated dementia[Title/Abstract]) OR (own syndrome[Title/Abstract] OR Alzheimer's disease[Title/Abstract]) OR (Alcohol related dementia[Title/Abstract]) OR (Frontotemporal dementia[Title/Abstract]) OR (Lewy Body Disease[Title/Abstract]) OR (Vascular dementia[Title/Abstract]) OR (alzheimer^*^[Title/Abstract]) OR (alzheimer[MeSH Terms])
2. (writing[MeSH Terms]) OR (writing^*^[Title/Abstract]) OR (handwriting[MeSH Terms]) OR (handwriting^*^[Title/Abstract]) OR (calligraphy[MeSH Terms]) OR (calligraphy[Title/Abstract])
3. “Review”[Publication Type] OR “Review Literature as Topic”[MeSH] OR “Systematic Review”[Publication Type] OR “Systematic Reviews as Topic”[MeSH] OR “Meta-Analysis”[Publication Type] OR “Meta-Analysis as Topic”[MeSH] OR “Network Meta-Analysis”[MeSH]
4. #1 AND #2 NOT #3 Filters: from 1991 - 2023

The terms placed in brackets indicate whether the phrase is a search keyword or a Medical Subject Heading (MeSH). All search queries were analyzed for registration as MeSH keywords.

A search was conducted to see whether it was necessary to include the search word “dementia” as a MeSH term with explosion and as a search query, as specified.

This was used to simplify the search term while preserving identical search results since specific databases had difficulties processing extensive search phrases. Comparable experiments were conducted across all datasets.

### 2.4 Data screening

All references were entered into EndNote 21, where duplicates, non-English entries, and irrelevant types not aligned with the article titles were detected and removed.

### 2.5 Data charting process

A reviewer cooperatively developed a data-charting form to determine the variables to extract. The reviewer independently extracted data and analyzed the results in the case of a disagreement. Demographic data from articles (author, year, nation, journal, and citations) and population characteristics (diagnosis, comorbidities, covariants, and disability) were retrieved, along with sample size (n), mean age, and sex distribution. The conclusion of data extraction was based on the research criteria of intervention, result, and effect. The studies examined the effects of writing in dementia, as well as the positive impact of writing in dementia, the Positive effects of writing in cases other than dementia, and sex differences. The knowledge and information levels of individuals were assessed.

### 2.6 Quality assessment

The bias risk was assessed using methodologies from the Cochrane Collaboration Risk of Bias Tool (CCRBT) and the Effective Public Health Practice Project Quality Assessment Tool to evaluate the methodological quality of primary studies employing various research designs (25). The six assessed components are: (1) selection bias, (2) research design, (3) confounders, (4) blinding, (5) data collection procedures, and (6) withdrawals and dropouts. Each component is classified as strong, moderate, or weak. In the absence of weak ratings, the overall rating is considered strong; one weak rating yields a middling classification, while two or more weak ratings result in a weak overall classification. The tool was modified as the component domain' confounders' is particularly relevant to randomized controlled trials, where controlling group differences is crucial. In the present study, populations served as their controls, diminishing the significance of this element. Two distinct evaluators assessed the research and reconciled any inconsistencies through dialogue.

## 3 Results

By carefully reviewing the 21 included articles which had been met out a topic, we found physical activities and cognitive activities such as writing, reading books or newspapers, poetry writing, journal writing, Chinese calligraphy writing, drawing, memory notebooks, crossword puzzles, board games or cards, participating in organized group discussions, and playing musical instruments have been mentioned frequently as an empowering tool to reduce the risk of dementia, improve cognitive function and even regulating emotions (26–45) (Table 1).

**Table 1:** Characteristics and main findings of studies included in the systematic review.

**Reference**	**Year**	**Country**	**Age range**	**Sample size**	**Diagnosis**	**Other comorbidities**	** Disability**	**Other covariants**	**Cognitive activity**	**Main finding**	**Positive effects of writingin dementia**	**Positive effects of writing in cases other than dementia **	**Sex difference**	** Knowledge/awareness**
				**Male/female/gender (%)**					**Physical activity**					
Platel et al. (38)	1993	England	–	22	AD	–	Writing impairments, graphomotor deficits, phonological and lexical impairments.	MMSE	Writing tests from dictation, including regular, irregular words, and non-words.	Agraphia in AD evolves from lexical errors to phonological and grapho-motor impairments.	Writing is preserved partially in mild dementia; phonological processing is relatively intact early.	–	–	Highlighted phases of impairment evolution: lexical, phonological, and grapho-motor decline.
				–					–					
Schmitter-Edgecombe et al. (41)	2008	USA	63–85	5	VMD	–	Memory impairment without significant functional decline; participants retained independence in daily activities.	Education level (mean 18 years), spousal support, and prior memory aid use.	Memory notebook training focused on prospective and retrospective memory tasks.	Memory notebook intervention improved memory strategy use and confidence in obtaining social support.	Enhanced memory strategy use, improved memory task performance, and emotional support.	–	Male caregivers benefited from reduced depression.	Participants and caregivers gained better-coping strategies and improved memory task performance.
				20–80					–					
Ryan et al. (39)	2009	Canada	–	9	Dementia, including AD	–	Cognitive and social impairments associated with dementia.	Social identity, personal relationships, and societal perceptions.	Writing and storytelling.	Highlights writing as a tool for empowerment, fostering emotional wellbeing, and challenging stereotypes of dementia.	Writing fosters social identity reclamation, emotional empowerment, and a sense of purpose, aiding individuals in processing and expressing their experiences.	Writing offers therapeutic benefits such as emotional clarity, stress reduction, self-discovery, and enhanced mental health through expressive outlets.	–	Writing increases awareness about personal experiences and societal misconceptions.
				–					–					
Johnson (72)	2009	US	79–85	4	AD	–	Cognitive impairment due to AD.	Memory notebook use, interaction frequency with staff and family.	Participants engaged in memory notebook use during group and individual sessions.	Memory notebooks reduced anger, agitation, and confusion and improved activity participation.	–	–	–	Caregivers and family gained insights into residents' needs and history through memory notebooks.
				25–75					–					
Crane et al. (53)	2009	USA	71–93	3,139	Dementia, AD, and VD.	Hypertension, diabetes, and stroke were evaluated as potential risk factors.	Cognitive decline was assessed via the CASI and neuropsychological testing.	APOE-e4 genotype, education level, head circumference, income, and BMI.	Literacy in Japanese and English was analyzed for associations with cognitive outcomes.	Proficiency in written Japanese was not protective against dementia.	–	–	–	Literacy and language usage were associated with cultural and educational background, not dementia prevention.
				100% male					–					
Kidd et al. (31)	2011	England	41–80	20	Dementia	–	–	–	Poetry writing intervention.	Poetry writing provided caregivers with self-affirmation, catharsis, and emotional relief.	Poetry writing helped caregivers reflect, gain empathy and acceptance, and release stress.	–	Female caregivers reported higher burden and depressive symptoms compared to males.	Poetry writing affirmed caregivers' roles, increased reflection, and promoted emotional growth.
				15–85					–					
Kwok et al. (35)	2011	New Zealand	70 years or older	31	Mild cognitive impairment.	–	Mild cognitive impairment affecting memory, orientation, and calculation.	Education level, prior calligraphy experience, baseline cognitive scores, age, and gender.	Calligraphy therapy (30 min/day, 5 days/week for 8 weeks) improved memory, attention, and orientation.	Calligraphy therapy significantly improved cognitive function (CMMSE score) compared to the control group.	Increased orientation, attention, calculation, and memory experience reduced cognitive decline.	–	–	Calligraphy was recognized as a feasible and culturally relevant cognitive therapy for older Chinese adults.
				21–79					–					
Petrescu et al. (37)	2014	Australia	–	4	–	–	–	–	Poetry writing intervention.	Poetry writing provided caregivers with self-affirmation, catharsis, and emotional relief.	Poetry writing helped caregivers reflect, gain empathy and acceptance, and release stress.	Benefits included catharsis, creativity, and self-awareness for other populations.	–	–
				100% female					–					
Thiel and Conroy (42)	2014	UK	55–74	4	Stroke, with additional characteristics of central linguistic deficits.	Severe aphasia or expressive difficulties.	All participant had acquired impairments from strokes, affecting their written and sometimes verbal communication.	Aphasia, apraxia of speech, and cognitive impairments post-stroke.	Therapy focusing on spelling and writing accuracy as part of their rehabilitation.	Both errorless and errorful therapies effectively improved spelling accuracy in individuals with acquired dysgraphia, with sustained effects at follow-up.	Writing, especially errorless methods, improves retention by reinforcing correct orthographic representations without confusion from errors.	Writing therapy (errorless or errorful) enhances spelling accuracy and speed in individuals with dysgraphia and aphasia.	–	Awareness of errors and the ability to self-monitor were critical factors influencing therapy success.
				75–25					–					
Blasko et al. (26)	2014	Austria	75 years	399	Non-demented individuals, MCI, and dementia.	Stroke and cardiac infarction.	Cognitive impairment progressing to dementia.	Gender, years of education, APOE-e4 allele presence, depressive symptoms.	Reading books, writing letters, and cognitive leisure activities.	Physical, cognitive, and social leisure activities reduce dementia risk and improve executive and memory functions.	Writing letters linked to lower conversion rates and improved cognitive function.	Reading and writing were protective against cognitive decline in older adults.	Males engaged more in physical activities; females participated more in cognitive activities.	Higher education is correlated with increased leisure activity engagement and improved cognitive resilience.
				–					Hiking, swimming, and dancing.					
Santos et al. (40)	2015	Brazil	60 years and older	97	Mild/Moderate AD and CIND	–	Cognitive impairment (mild to moderate AD, CIND).	Education level, baseline MMSE, and GDS scores.	Reading, memory training, speech therapy, logic games, and art therapy were included.	Multidisciplinary rehabilitation improved cognition quality of life, and reduced depression in mild AD and CIND groups.	Writing tasks enhanced cognitive stimulation in mild AD participants during rehabilitation.	–	Female participants were more prevalent in moderate AD and CIND groups; sex differences were not analyzed.	Participation improved awareness and social engagement among mild AD and CIND groups.
				31–69					Physical therapy and training sessions.					
Chan et al. (27)	2017	Hong Kong	60–85	99	MCI	–	–	Education level, type of MCI (amnestic vs. non-amnestic), baseline cognitive performance.	Chinese calligraphy writing training, focusing on visual encoding and mnemonic strategies.	Calligraphy training significantly improved working memory and attentional control, with some long-lasting effects.	Enhanced memory and attention; fostered mental and motor skills integration.	Benefits observed in enhancing creativity, stress reduction, and emotional stability.	–	Participants became more aware of cognitive and attentional strategies via task-focused practice.
				33–67					–					
Kurita et al. (33)	2020	Japan	70 years and older	2,668	Normal	Diabetes, heart disease, respiratory diseases, and chronic pain.	Assessed as needing support or care using Japan's Long-Term Care Insurance system criteria.	Gait speed, BMI, education, depressive symptoms, and chronic diseases were included as covariates.	Activities included writing for pleasure, reading, board games, puzzles, and musical activities.	Writing was not directly studied; it was part of cognitive activities associated with reduced disability risk.	Writing for pleasure was part of high CA linked to better outcomes in older adults.	–	–	Combined PA and CA engagement showed synergistic benefits for reducing disability risks.
				51.6–48.4					Moderate-to-vigorous physical activity.					
Kurita et al. (34)	2020	Japan	70 years and older	2,726	Cognitive impairment.	Chronic diseases (hypertension, diabetes, hyperlipidemia, heart disease).	–	Education, body mass index, chronic diseases, medication use, depressive symptoms, gait speed, smoking, drinking, and employment.	Writing, reading, puzzles, board games, discussions, and music.	High physical and cognitive activity showed additive protective effects against cognitive impairment.	Writing was part of cognitive activities associated with lower odds of impairment; effects specific to dementia were not detailed.	Writing reduced cognitive impairment risk.	–	Promoted awareness of the synergistic benefits of combining physical and cognitive activities.
				48.2–51.8					Moderate-to-vigorous activity.					
Feng et al. (28)	2020	China	55 years and older	8,326	MCI	–	–	Education, BMI, smoking, drinking, marital status, hypertension, diabetes, and income.	Writing or reading.	Reading and writing reduced MCI risk; TV/computer use and unhealthy foods increased MCI risk.	Not directly assessed for dementia; writing reduced MCI incidence, a precursor to dementia.	Writing fostered cognitive engagement and reduced MCI risk.	Health behaviors were more protective in males; obesity patterns were more protective in females.	Promoted understanding of modifiable behaviors, like reading and writing, to lower MCI risk.
				46.9–53.1					Martial arts, ping pong, and aerobic exercises.					
Krajenbrink et al. (32)	2020	Australia	60	1	svPPA, semantic dementia	–	–	–	Repetition, reading, and writing tasks using lexical retrieval therapy and conceptual enrichment.	Writing improved lexical retrieval; combined spoken and written therapy had a more significant impact than spoken alone.	Enhanced semantic connections, improved word comprehension, and better writing accuracy.	Potential for improving word retrieval in non-dementia aphasia patients with tailored therapy.	–	Participants gained insight into compensatory strategies; therapy demonstrated limited generalization outside sessions.
				100% male					–					
Meade et al. (45)	2020	Canada	73–91	56	MCI	–	Dementia-related memory impairment; reduced visuospatial and verbal fluency in some participants.	Cognitive and visuospatial abilities, MoCA/MMSE scores, and Rey-Osterrieth figure copying.	Tasks included drawing, writing, and memory recognition.	Drawing improves memory more than writing in older adults, including dementia patients.	Drawing engages preserved visual regions, improving memory recall.	Drawing enhances memory by integrating visual and semantic information.	–	Participants were largely unaware of the superior efficacy of drawing over writing for memory enhancement.
				32.5–67.5					–					
Tsuda et al. (43)	2022	Japan	50–89	8	Dementia (ranging from very mild to moderate severity).	Vascular issues or recurrent non-convulsive seizures.	Cognitive impairments affect memory, social behaviors, and psychological conditions.	Duration of journal writing, living arrangements, and daily routines.	Journal writing	Journal writing supports self-reflection, identity reaffirmation, recovery, and mental organization while highlighting the potential adverse effects of pessimistic reflections.	Improves self-awareness, promotes emotional recovery, aids memory, and helps maintain a sense of identity.	–	–	Journal writing fosters a better understanding of personal history and enhances caregivers' insights into participants' challenges and achievements.
				50–50					–					
Malcorra et al. (36)	2022	Brazil	51–82	117	Healthy adults without dementia.	No comorbidities were reported.	None; cognitive function within the normal range (assessed by MMSE).	Education, socioeconomic status, RWH.	Reading and writing habits.	Higher education and frequent RWH improved macro- and microstructural aspects of oral discourse.	–	Enhanced oral discourse and linguistic coherence in typical adults.	–	Frequent RWH and higher education build cognitive reserve, delaying potential cognitive decline.
				–					–					
Hsiao et al. (29)	2023	Taiwan	62–82	30	MCI	–	Cognitive impairments, reduced attention, emotional instability, and motor skill challenges.	Education years, cognitive baseline scores, emotional state.	CCH involves visual perception, spatial structures, and planning.	Calligraphy training improved cognition, emotion, attention, motor coordination, and language.	Enhanced memory, emotional stability, attention, and motor skills.	Benefits in cognitive and emotional improvements for similar age groups without MCI.	–	Participants developed mindfulness, improved focus, and emotional regulation through consistent practice.
				23–77					CCH involves clearing all clutter, relaxing the body and sitting up straight, holding the upper arm at a precise angle, lifting the calligraphy pen, lightly lifting with an inhalation, and pressing the pen to the paper with an exhalation.					
Wu et al. (44)	2023	Australia	70 years or older.	10,318	Dementia	Diabetes, hypertension, and depression.	Cognitive decline.	The models adjusted education level, socioeconomic status, physical activity, and frailty.	Writing letters, journaling, puzzles, and education classes.	Cognitive and literacy activities reduced dementia risk by up to 11%.	Improved cognitive reserve, delayed onset of symptoms.	Enhanced cognitive resilience and mental engagement in older adults.	No significant differences except creative artistic activities showed marginal benefits for men.	Literacy activities support dementia prevention and cognitive reserve development.
Reference	Year	Country	Age range	Sample size	Diagnosis	Other comorbidities	Disability	Other covariants	Cognitive activity	Main finding	Positive effects of writingin dementia	Positive effects of writing in cases other than dementia	Sex difference	Knowledge/awareness
				Male/female/gender (%)					Physical activity					
				47.4–52.6					Moderate to vigorous physical activity.					

AD, Alzheimer's Disease; APOE-e4, Apolipoprotein E epsilon 4 allele; BMI, Body Mass Index; BPSD, Behavioral and Psychological Symptomes of Dementia; CA, Cognitive Activity; CASI, Cognitive Abilities Screening Instrument; CCH, Chinese Calligraphy Handwriting; CIND, Cognitive Impairment Without Dementia; GDS, Geriatric Depression Scale; MCI, Mild Cognitive Impairment; MMSE, Mini-Mental State Examination; MoCA, Montreal Cognitive Assessment; PA, Physical Activity; RWH, Reading and Writing Habits; svPPA, Semantic Variant of Primary Progressive Aphasia; VD, Vascular Dementia; VMD, Very Mild Dementia.

We have categorized our findings into 6 main classes to provide a better understanding of this topic.

### 3.1 Cultural and artistic writing practices

Chinese calligraphy therapy (CCT) is a branch of art therapy involving culture, health, behavioral treatment, and rehabilitation for patients with cognitive impairments. CCH performance integrates the mind and body and involves visual perception, spatial structures, and planning (46, 47). The practice of CCH requires relaxing the body and sitting up straight, holding the upper arm at an accurate angle, lightly lifting with an inhalation, and pressing the pen to the paper with an exhalation while moving through the order of brush strokes step by step (48).

Previous studies have suggested that Chinese calligraphy practice can have many advantages on cognitive functions as well as Chan et al. (27) and Kwok et al. (35) found that Chinese calligraphy writing mainly involved visual encoding, memory rehearsal, and attentional control, which significantly improved specific cognitive functions, especially working memory, orientation, attention, and calculation; participants experienced reduced cognitive decline. Therefore, Chinese calligraphy writing was demonstrated to serve as an effective non-pharmacological intervention in participants with Mild Cognitive Impairment (MCI). They also mentioned that CC was recognized as a feasible and culturally relevant cognitive therapy for older Chinese adults, which is crucial for using culturally relevant therapies for older adults.

Moreover, Hsiao et al. (29) conducted a CCH training program, which additionally included oral presentations of the works of each participant. They showed Chinese calligraphy training courses can also help with visuospatial memory, emotion regulation by persistent happiness and looking forward to practicing time, upper limb coordination, language, and deep thinking, improved Speech function through oral presentations, improved writing speed and accuracy, and abstract thinking. Since the training was performed through breathing control, it can also modulate parasympathetic nerves, stabilize mental states, lower respiration and heart rate, and reduce blood pressure.

There are many types of arts, artistic writings, and cognitive activities that were frequently suggested in surveyed studies that can have protective effects against cognitive impairment, reduce the risk of disability onset, and improve executive and memory functions, attention, and cognitive reserve; these activities included reading books or newspapers, writing for pleasure, story-telling, also doing crossword puzzles, playing board games or cards, playing musical instruments and speech therapy (26, 28, 33, 34, 36, 39, 40, 44).

Particularly, Meade et al. (45) discovered that drawing significantly enhanced memory more than writing in healthy individuals with probable dementia. This might result from the weaker memory encoding provided by writing compared to drawing. They also stated that writing engages residual verbal and motor functions, improving some episodic memory in mild cognitive impairments; however, in dementia, due to impaired visual-perceptual brain regions, writing was less effective than drawing.

### 3.2 Poetry and creative writing as therapeutic tools

Poetry and creative writing have been proven to include significant advantages for cognitive functions. As Petrescu et al. (37) demonstrated, poetry writing workshops improved the conceptions of personhood by motivating the feeling of competence and self-efficacy. This also resulted in overcoming a prevailing stereotype that dementia equates with dependency. Furthermore, their study also showed that poetry writing helps with understanding the experience of dementia, stimulating the participants' creativity, identifying potential talents, and showing a drive for personal growth, which is still present in people with dementia.

Another study figured out that even poetry writing in caregivers of patients with dementia can have many positive impacts, such as psychological benefits, Self-Affirmation, improved sense of achievement, greater acceptance, empathy, self-awareness, reflection, positive challenge, and helping others, all of them can result in enhancing understanding of their roles and the challenges they face (31).

Journaling and storytelling are other therapeutic tools that can help with different aspects of dementia. Journal writing was suggested by Tsuda et al. (43) to Improve self-awareness, promote emotional recovery, aid memory, and help maintain a sense of identity; however, it intensified regrets and pessimistic emotions due to reviewing past independence or losses. Moreover, they discovered that journal writing enhanced caregivers' insights into participants' challenges and achievements. Another study by Ryan et al. (39) also stated that writing and story-telling foster emotional clarity and stress reduction, which were provided by expressing their experiences. Despite this, the study showed writing may reinforce feelings of frustration when cognitive impairments negatively affect the ability to express thoughts effectively; also, writing about traumatic events can initially cause emotional distress and rumination in some individuals.

Memory notebook training has also been mentioned as having potential benefits for cognitive impairments and Alzheimer's disease. Johnson (30) studied the effectiveness of Memory Notebooks in patients with Alzheimer's Disease; they concluded Memory notebooks reduced anger, agitation, and confusion and encouraged participation in activities; they also found Caregivers and families gained insights into residents' needs through these memory notebooks. Another study about assessing memory notebook intervention for Memory-impaired individuals without major functional decline was conducted by Schmitter-Edgecombe et al. (41) and resulted in enhanced memory strategy use, improved memory task performance, and emotional and confidence in obtaining social support; additionally, caregivers gained better coping strategies.

Writing letters is another type of writing that links to lower dementia conversion rates and improved cognitive function (26).

### 3.3 Writing to reaffirm identity and social roles

Selfhood is equated with memory and language (49), autobiographical memories (50), and through the accounts of caregivers (51); therefore, selfhood gets lost in dementia. Therefore, there is a significant need to reconstruct and reaffirm identity and social roles in dementia since Cohen-Mansfield et al. (52) also provided clear evidence that treatment aimed at strengthening the residual self-identities, favored roles, and personal attributes of elderly persons with dementia can improve their wellbeing.

Journal writing is one of the activities that appeared to support self-reflection, identity reaffirmation, recovery, and mental organization (43). Storytelling writing can also empower social identity reclamation and create a sense of purpose in individuals (39).

As Petrescu et al. (37) found that poetry writing can also be used as a tool to maintain and reinforce a sense of self-identity.

### 3.4 Impacts of structured writing interventions

In the study conducted by Krajenbrink et al. (32), they surveyed treatments for spoken and written word recall in primary progressive aphasia, in which they evaluated the efficacy of two treatments of Repetition and Reading in the Presence of a Picture (RRIPP) with and without required written responses, and procedures of Conceptual Enrichment (COEN). COEN treatment did not result in significant gains in word retrieval or comprehension; however, RRIPP led to considerable improvement of treated items on a comprehension task, improvements in spoken and written word retrieval, improvement in naming, and improved lexical retrieval, although more significant improvement got demonstrated when written production was required.

Thiel and Conroy (42) conducted a study on patients with acquired written and sometimes verbal communication impairments from strokes. They performed errorful and errorless therapies that focused on spelling and writing accuracy as part of their rehabilitation. The study demonstrated that writing therapy (errorless or errorful) enhanced spelling accuracy and speed. Writing, especially errorless methods, improved retention by reinforcing correct orthographic representations without confusion from errors; however, errorful therapy showed negative influences on spelling. They also concluded that awareness of mistakes and self-monitoring influenced therapy success.

On the other hand, despite previous studies, Crane et al. (53) found Proficiency in written Japanese had no protective effects against dementia, and literacy and language usage were associated with cultural and educational background, not dementia prevention.

### 3.5 Evolution of writing skills in dementia

Platel et al. (38) studied the evolution of writing impairment in Alzheimer's disease; they found agraphia in Alzheimer's disease follows a logical development including three phases: moderate impairment, prolonged impairment characterized by non-phonological spelling mistakes, and severe impairment accompanied by considerable concerns in graphic motor function. Moreover, patients with Alzheimer's disease have shown a restricted level of awareness of their mistakes.

### 3.6 Writing as a multimodal rehabilitation component

Multidisciplinary rehabilitation, including reading, writing letters, memory training, speech therapy, logic games, art therapy, and physical therapy, were proven to have positive influences on cognition, quality of life, and reduced depression in Mild/Moderate Alzheimer's Disease (AD) and Cognitive Impairment Without Dementia (CIND) (26, 40, 44).

Writing tasks enhanced cognitive stimulation in mild AD participants as a part of the rehabilitation (40).

Kurita et al. (33, 34) suggested that engaging in both physical activities (PA) and cognitive activities (CA) is more effective in reducing the risk of disability onset than engaging in either PA or CA alone and demonstrated High physical and cognitive activity showed additive protective effects against cognitive impairment and synergistic benefits of combining physical and cognitive activities.

Therefore, Raising awareness about the importance of maintaining an active and engaged physical and cognitive lifestyle is crucial to reducing the risk of cognitive decline and dementia (28).

### 3.7 Sex differences

The impact of cognitive and physical activities might vary between sexes in terms of cognitive health (28), and it has also been suggested that males engaged more in physical activities; females participated more in cognitive activities (26).

### 3.8 Educations

Higher education has been found to help with increased leisure activity engagement and improved cognitive resilience (26). Malcorra et al. (36) indicated that higher education and frequent reading and writing habits (RWH) in individuals with normal cognitive function could improve macro- and micro-structural aspects of oral discourse linguistic coherence, build cognitive reserve, and delay potential cognitive decline.

## 4 Discussion

After carefully reviewing 12 studies on this subject, we determined that the most mentioned benefits of writing in people with dementia were significant improvements in working memory, attentional control, visuospatial memory, word retrieval, and overall cognitive resilience.

Writing by hand has been found to cause a wide variety of beneficial effects on brain functioning, for instance, improvement of memory, recall of words, and connectivity within the dorsal attentional network (54–56). Writing can cause significant improvements in the rehabilitation of people with cognitive impairment and dementia (35). Additionally, the remarkable advantages of calligraphy in dementia have been proven, and calligraphy therapy has been considered the most effective art therapy for improving cognitive function (4). This outcome may be because calligraphy integrates visual performance with spatial abilities and cognitive planning, which helps with a better cognitive function, and it also can improve concentration, orientation, calculation, controlling the body, emotional stability, and eventually quality of life (35, 57, 58). Japanese calligraphy has positively affected relaxation, mindfulness, depression, emotional regulation, and mental health in older adults (59) which can be concluded to have indirect impacts on cognitive improvements.

In Iran, Persian calligraphy is being taught in early elementary school. This activity demands coordination of the upper limb, visuospatial domains, working memory, and concentration to perform the curves, edges, strokes, and correct spelling. Thus, for Persian-speaking elderly with or without cognitive impairments, Persian calligraphy could be considered a magnificent therapy for their rehabilitation.

No studies have evaluated the impact of writing in a specific language as cognitive rehabilitation and have compared it with other languages. However, due to the various cognition levels involved in different languages (60), further evaluation is suggested around this subject in the future.

We also noted that Handwriting as a rehabilitation tool, particularly the types of writing through which individuals could express their emotions, thoughts, and experiences, such as journaling, storytelling, and creative writing, will significantly improve the emotional wellbeing through reducing anxiety, depression and self-awareness of patients with MCI or dementia which help maintain a sense of social identity and communication with others. As Social isolation and lack of social activity have been highly identified with an increased risk of dementia (61–63), and social interactions that provide feelings of satisfaction and perceived reciprocity have demonstrated preventive effects toward dementia (64).

However, nowadays, digitalization has affected every aspect of our lives, such as banking, purchasing, learning, and significant communications that have become mostly through social networks; digital writing by computers, tablets, or mobile phones is increasingly replacing writing by hand (65, 66). Adults mostly use typing to produce written texts such as messages, letters, or notes in everyday or professional life instead of Handwriting. Writing on paper with a pen or pencil has become old-fashioned and uncommon. Unfortunately, due to these changes, people cannot benefit from writing on paper Traditional Handwriting by pen and paper significantly impacts cognitive rehabilitation and has been demonstrated to have many advantages over typing on cognition and memory (18, 67). Since typing and Handwriting activate the brain with different patterns, typing contributes to less activation (68), functional magnetic resonance imaging also demonstrated that Handwriting activates a broader network of brain regions involved in motor, sensory, and cognitive processing compared to typing, which engages fewer neural circuits and lowers cognitive engagement (69). Moreover, many studies have suggested Handwriting over typing, for example, typing has been associated with impaired learning in students due to the shallower processing (17), Handwriting showed enhancements in brain connectivity, not typing (18).

Some evidences support the benefits of computer-based interventions as a rehabilitation in patients with dementia and cognitive impairments (70), such as significant improvements in delayed, working, and short-term memory and in language abilities (71), But not particularly mentioned typing.

There was a crucial need to reflect on the benefits of writing on paper as a potential rehabilitation therapy for dementia to encourage and motivate patients and their caregivers to pay more attention to this area and to get healthcare systems to consider writing therapy as a part of routine rehabilitation therapies. Therefore, in this study, we assessed the potency of writing as a part of rehabilitation in people with cognitive impairments by reviewing previous studies.

We aim to raise awareness about the importance of physically and cognitively maintaining an active life in people with dementia by providing an accessible and affordable solution. Writing as a rehabilitation therapy has these characteristics and can be done quickly by anyone, even with physical or mental limitations. Also, using culturally relevant cognitive activities is noticeable for older adults to get better results, as calligraphy therapy in Taiwanese individuals, which caused a sense of nostalgia and wealth of sensory stimulation since they had been taught Chinese calligraphy during early school life in Taiwan (29).

## 5 Conclusion

We found that writing has benefits other than cognitive improvements, such as positive effects on mental health, increased coordination of upper limbs, and higher self-confidence.

Eventually, by gathering all this information and proving results, we suggest writing on paper with a pen or pencil as an old-fashioned way to be a part of rehabilitation therapy for people with dementia and even healthy older adults, as it has preventive effects toward cognitive impairments. If writing therapy is done, creative writing can have more privileges, such as enabling self-expression and communication, and promoting positive self-worth.

## Data Availability

The original contributions presented in the study are included in the article/Supplementary material, further inquiries can be directed to the corresponding author.
